# Midwifery

**Published:** 1836-04

**Authors:** 


### MIDWIFERY.

### *On the Reposition of the Umbilical Cord.* By Dr. MICHAELIS, of Kiel.

[WE have not read an obstetrical essay in a periodical work for some time which has given us more pleasure. Dr. M. has examined the subject with great attention, and has given an admirable exposition of the circumstances under which prolapsus of the funis takes place, and of the means adopted by nature to prevent it. Hitherto it had been always considered as useless to attempt returning a prolapsed umbilical cord; the coil slips down as fast as it is pushed up; nor have the various plans which have been recommended (and which are too well known to the scientific obstetrician to require enumerating,) proved at all more successful. We can only find space for a brief outline of this memoir, which we shall give partly in the words of the author and partly in our own.]

The most effectual means by which we can hope to keep back the umbilical cord after it has been replaced, is a permanent contraction of the uterus round the presenting part of the child, which either comes on of itself in every labour, or where this is not the case,—viz. where there is a large quantity of liquor amnii, or not much uterine activity,—may be most certainly induced by mechanical irritation. The portio vaginalis is that part of the uterus which first contracts round, or rather applies itself to, the child's head: the effect of this action is to push away the cord which lies near the head shortly before the os uteri begins to dilate; but, where this contraction does not take place, because the head is still high when the membranes rupture, or because it comes down irregularly, the cord is very apt to prolapse when the liquor amnii comes away. As, however, the umbilical cord is seldom noticed until the rupture of the membranes, or until the os uteri is nearly fully dilated, this state of the portio vaginalis, as far as regards its reposition, is of less consequence. On the other hand, the contraction of the uterus, which comes on with the rupture of the membranes, and sometimes where they protrude very much even before, is of great importance. This contraction takes place in the inferior segment of the uterus: it surrounds the head, and, when fully developed, extends over the whole head of the child. Thus, for instance, if we attempt to operate at an early stage, it feels more like a hard ring round the head, of about a finger's breadth, and it may be felt to extend itself higher up in proportion as the stimulus of the hand excites the activity of the uterus. There are two circumstances in this contraction which especially deserve notice: 1st. that it always rises from below upwards, and, when once established, continues during the intervals of the pains. As soon as the os uteri is nearly fully dilated, it begins to form, and, according to Dr. M.'s observations, continues until the head has quitted the uterus. 2d. That its upper edge is well defined, and does not gradually pass into the other portions of the uterus. The action of this stricture (if we may give it such a name,) as respects the cord, is very distinct: where the cord has laid rather low down, it pushes it back, and keeps it in this situation, so long as the presenting part of the child is round, viz. the head or nates. This stricture is alone the means to which the accoucheur must look for assistance, if he wishes effectually to replace the cord; a means which he must use if he finds it present, and which, if it be not present, he can easily induce by slight irritation of the uterus. It is a great fault, in former, observations on this subject, that the pelvis has always been looked upon as the means for keeping up the cord when returned, and little or no attention paid to the uterus. It is the uterus alone which prevents the cord from prolapsing, and to which the operator must look for assistance if he intends to succeed: hence the proposition to push up a sponge so as to fill the empty space between the head and pelvis is futile, and has long been forgotten.

From what has been stated, reposition of the cord consists in carrying it above that circular portion of the uterus which is contracted over the presenting part. The reposition of the cord may be effected by the hand, or by means of an elastic catheter and ligature. In replacing the cord by means of the hand alone, Dr. Michaelis remarks that we shall effect this more readily by merely insinuating the hand between the head and the uterus, and gradually passing it further round the head, pushing the cord before it: in this manner we do not require to rupture the membranes where we have felt the cord before the liquor amnii has escaped; a point of considerable importance.

The reposition by means of the catheter is effected by passing a silk ligature, doubled, along a stout thick elastic catheter, from twelve to sixteen inches in length, so that the loop comes out at the upper extremity; the catheter is introduced into the vagina, and the ligature is passed through the coil of umbilical cord, and again brought down to the os externum. A stilet with a wooden handle is introduced, and its point passed out at its upper orifice, and the loop of the ligature hung upon it: it is then drawn back into the catheter, and pushed up to the end. The operator has now only to pull down the ends of the ligature, passing the catheter up to the cord, which now becomes securely fixed to its extremity. When the reposition has been effected, he has merely to withdraw the stilet; the cord is instantly disengaged. To prevent any injury, &c. the ligature should be brought away first, and then the catheter.

Dr. Michaelis has recorded eleven cases of prolapsus of the cord, where it has been returned by the above means, in nine of which the child was born alive. In three cases the arm presented also, which was replaced, and the head brought down: in two of these the child was born alive.

[These facts are very interesting, and deserve careful attention. The reposition of the prolapsed cord has long been a much wished-for object, and the possession of the means by which turning in many of these cases is rendered unnecessary will, of course, be a most valuable addition to obstetric knowledge.]

*Neue Zeitschrift für Geburtskunde,* III. *Band.* 1 *Heft.* 1835.

---

*Tables of the Midwifery Cases in the Maison d'Accouchement de Paris, from* 15 *June,* 1830, *to* 15 *June,* 1835.

*Table of Presentations.*

| Years. | Vertex | Face. | Nates. | Feet | Knees. | Right Shoulder. | Left Shoulder. | Total. |
|---|---|---|---|---|---|---|---|---|
| 6 months 1830 | 881 | 1 | 33 | 13 | .. | 2 | .. | 930 |
| 1831 | 2,714 | 6 | 26 | 11 | .. | 4 | 5 | 2,766 |
| 1832 | 2,198 | 8 | 67 | 19 | 1 | 4 | 6 | 2,303 |
| 1833 | 2,171 | 11 | 49 | 17 | 1 | 6 | 6 | 2,261 |
| 1834 | 2,289 | 9 | 56 | 20 | 1 | 11 | 2 | 2,388 |
| 6 months 1835 | 1,071 | 3 | 26 | 14 | .. | .. | 3 | 1,117 |
| General Total | 11,324 | 38 | 257 | 94 | 3 | 27 | 22 | 11,765 |

*Results.*

| Years. | Living. Single. | Living. Twins. | Dead. During Labour. | Dead. Putrid | Total. | Kind of Labours. Natural. | Kind of Labours. Artificial. | Kind of Labours. Laborious. | Total. |
|---|---|---|---|---|---|---|---|---|---|
| 6 months 1830 | 848 | 30 | 24 | 28 | 930 | 922 | 2 | 6 | 930 |
| 1831 | 2,623 | 44 | 45 | 54 | 2,766 | 2,744 | 9 | 13 | 2,766 |
| 1832 | 2,128 | 50 | 52 | 73 | 2,303 | 2,266 | 14 | 23 | 2,303 |
| 1833 | 2,078 | 49 | 57 | 77 | 2,261 | 2,219 | 13 | 29 | 2,261 |
| 1834 | 2,211 | 56 | 53 | 68 | 2,388 | 2,352 | 8 | 28 | 2,388 |
| 6 months 1835 | 1,031 | 34 | 19 | 33 | 1,117 | 1,108 | 4 | 5 | 1,117 |
| Total | 10,919 | 263 | 250 | 333 | 11,765 | 11,611 | 50 | 104 | 11,765 |

*La Lancette Française, No.* 115, 26 *Sept.* 1835.

